# Treatment of full-length ureteral avulsion due to ureteroscopy: Two case reports and a review of the literature

**DOI:** 10.1097/MD.0000000000051335

**Published:** 2026-10-30

**Authors:** Bin Feng, Xiaopeng Wu, Yilin Yuan, Yongtao He, Xuesong Li, Guanghui Yan, Kunlin Yang

**Affiliations:** a Department of Urology, Gansu Provincial Hospital, Lanzhou, Gansu, China; b First Clinical Medical College, Gansu University of Chinese Medicine, Lanzhou, Gansu, China; c Department of Urology, Peking University First Hospital, Beijing, China; d The First Hospital of Lanzhou University, Lanzhou, Gansu, China.

**Keywords:** full-length ureteral avulsion, preventive measures, totally intracorporeal robot-assisted ileal ureter replacement, ureteroscopy

## Abstract

**Rationale:**

Full-length ureteral avulsion is one of the most severe complications of ureteroscopy. Although this condition is extremely rare, the absence of standardized treatment protocols poses significant challenges for urologists in clinical management.

**Patient concerns:**

In case A, the subject is a 48-year-old male, and in case B, the subject is a 63-year-old female. Both patients had full-length ureteral avulsion during ureteroscopic lithotripsy. Case B underwent emergent primary repair involving pyeloureterostomy, ureteroneocystostomy, and omental wrapping; however, the procedure failed to achieve satisfactory functional recovery, resulting in persistent obstruction.

**Diagnoses:**

Based on clinical history and imaging findings, case A was diagnosed with full-length ureteral avulsion, and case B presented with ureteral stricture following primary in situ repair of full-length ureteral avulsion.

**Intervention:**

Both patients were transferred to our center for upper urinary tract reconstruction 5 months after the original injury. Following bowel preparation, they underwent totally intracorporeal robot-assisted ileal ureter replacement.

**Outcomes:**

Both patients demonstrated stable total renal function and adequate drainage from the reconstructed renal units postoperatively.

**Lessons:**

The totally intracorporeal robot-assisted ileal ureter replacement represents a safe and effective treatment in managing full-length avulsion injuries of the ureter. Lifelong follow-up remains essential after reconstructive surgery and should include surveillance for obstruction, infection, metabolic complications, and renal deterioration. Preventive measures need to maintain paramount importance in clinical practice to avert such severe iatrogenic complications.

## 1. Introduction

Full-length ureteral avulsion represents a complete disarticulation of the ureter from the ureteropelvic junction (UPJ) to the ureterovesical junction. It is regarded as one of the most serious complications of ureteroscopy. Although rare, the consequences for both the patient and the surgeon involved can be severe and distressing. This article reports the management of 2 cases of full-length ureteral avulsion caused by ureteroscopy.

A literature search was performed in PubMed in 2026, using the following search strategy: “ureteral avulsion” OR “full-length ureteral avulsion” OR “ureteral avulsion,” AND “ureteroscopy” OR “ureteroscopy,” AND “treatment” OR “prevention” OR “management.” The search covered publications from 1988 to 2025. After screening titles and abstracts, we excluded non‑relevant articles, duplicates, and studies without full‑text availability. A total of 55 records were initially identified, 34 were screened, and 21 were included in this review. No additional reference‑list screening was performed.

## 2. Case presentation

### 2.1. Case A

A 48-year-old male patient underwent rigid ureteroscopic lithotripsy with a holmium laser for a left proximal ureteral calculus 5 months prior. During the procedure, difficulty in withdrawing the ureteroscope was encountered due to poor visualization following stone fragmentation. After forceful removal of the ureteroscope, severe hemorrhage at the left ureteral orifice prevented placement of a double-J stent. On the 1st postoperative day, the patient spontaneously expelled an approximately 28 cm segment of the avulsed ureter during voiding (Fig. 1A). Subsequently, a left renal pelvis drainage catheter was placed under ultrasound guidance to drain urine and prevent urinary extravasation. Preoperative imaging evaluation – computed tomography urography, comprising antegrade pyelography and volumetric composite imaging – demonstrated absence of the left ureter (Fig. 1B–D). The glomerular filtration rate (GFR) was measured at 43.4 mL/min for the left kidney and 49.7 mL/min for the right kidney.

**Figure 1.**
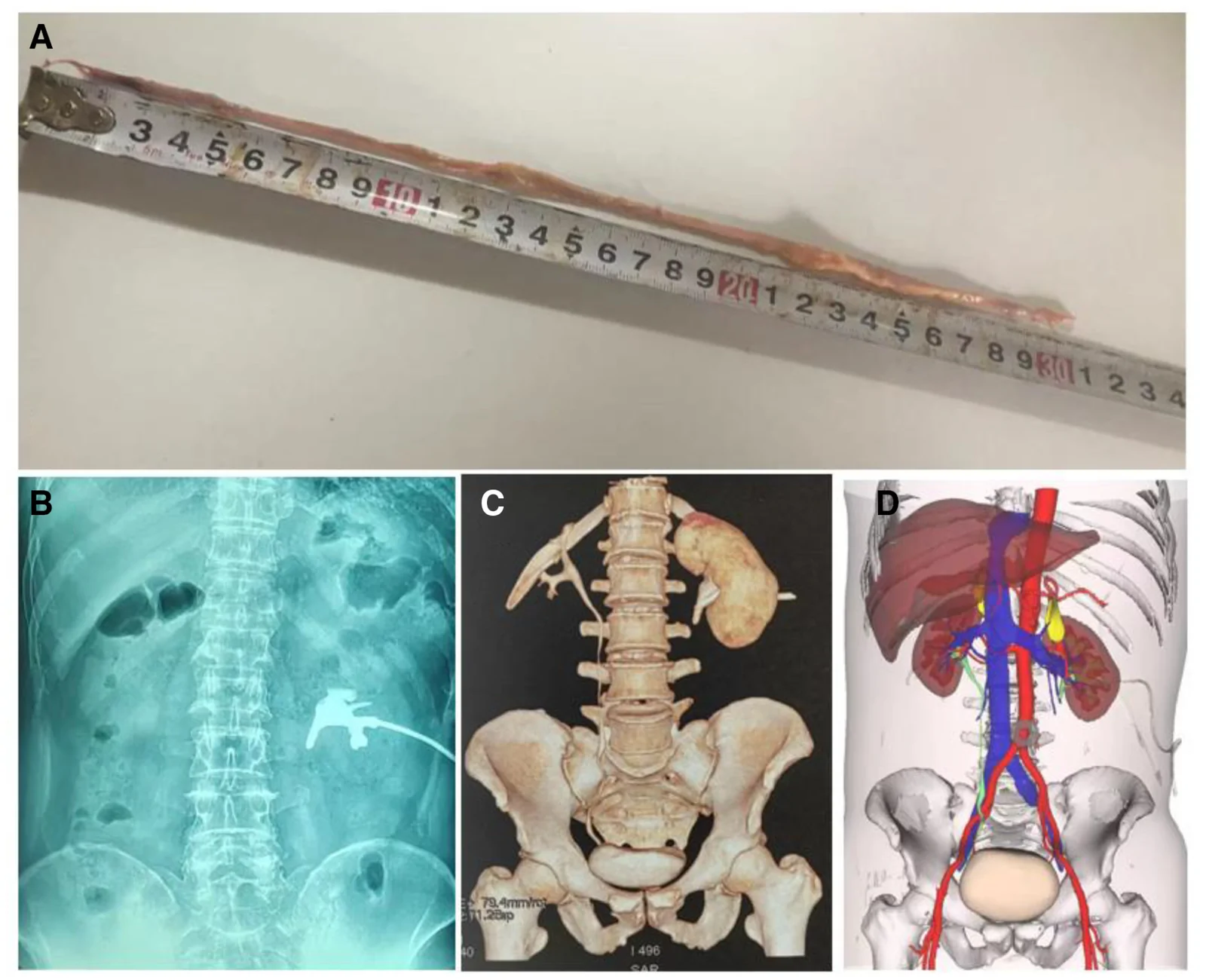
Case A: clinical data. (A) Full-length ureteral avulsion. (B–D) CTU, antegrade pyelography, and volumetric composite imaging demonstrated absence of the left ureter.

### 2.2. Case B

A 63-year-old female patient underwent flexible ureteroscopy with holmium laser lithotripsy for a right renal calculus 5 months prior. During the procedure, the flexible ureteroscope sheath caused full-length ureteral avulsion. The surgery was immediately converted to laparoscopic right pyeloureterostomy with ureterovesical anastomosis and nephrostomy. Intraoperatively, after the anastomosis of the avulsed ureter was completed, the ureter was wrapped with omentum to ensure adequate blood supply to the distal ureteral stump. Two months postoperatively, following removal of the double-J stent and clamping of the nephrostomy tube, the patient developed right flank discomfort. Further antegrade urography revealed a 5 cm stricture/obstruction in the distal ureter (Fig. 2B), accompanied by dysfunctional ureteral peristaltic activity. Preoperative computed tomography and volumetric composite imaging revealed atresia of the distal right ureter (Fig. 2C and D). The GFR was measured at 52.6 mL/min for the left kidney and 39.2 mL/min for the right kidney.

**Figure 2.**
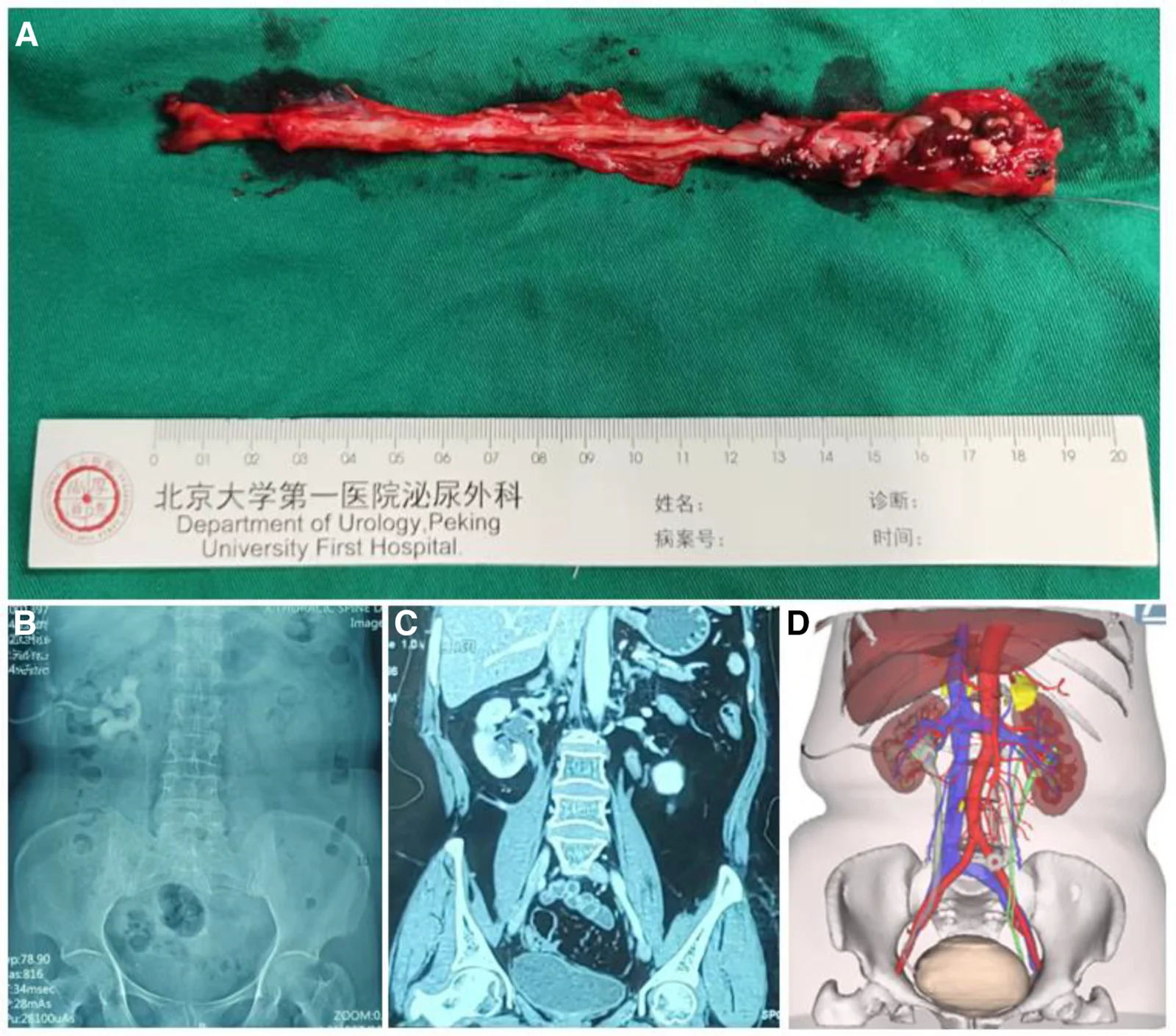
Case B: clinical data. (A) Surgically resected injured ureter. (B–D) CTU, antegrade pyelography, and volumetric composite imaging revealed atresia of the distal right ureter.

Both patients were transferred to our center for definitive upper urinary tract reconstruction. Following bowel preparation, they underwent totally intracorporeal robot-assisted ileal ureter replacement (RA-IUR): The proximal ureter and bladder were mobilized (with resection of the damaged ureter in case B (Fig. 2A)). A 25 cm ileal segment was harvested 20 cm proximal to the ileocecal valve, followed by restoration of intestinal continuity (Fig. 3A). The harvested ileum was intubated with a double-J stent, and an artificial nipple valve was fashioned. The ileal ureter was transposed through a mesenteric window into the retroperitoneal space (Fig. 3B) to reduce gastrointestinal complications, maintain the ileal ureter in a ureteral bed, and minimize internal hernia and adhesion-related risks. The ileal ureter was anchored with iliopsoas muscle suspension to prevent kinking and tension at the anastomoses. The ileal afferent limb was anastomosed to the renal pelvis (Fig. 3C). The ileal nipple valve was anastomosed to the incised dome of the bladder (Fig. 3D). The proximal and distal anastomoses, involving the renal pelvis, ileal ureter, and bladder, were performed intracorporeally using a continuous full-thickness suture technique. A wide anastomosis and adequate ileal segment length maintain the ileal ureter under low tension, thus ensuring optimal postoperative drainage.

**Figure 3.**
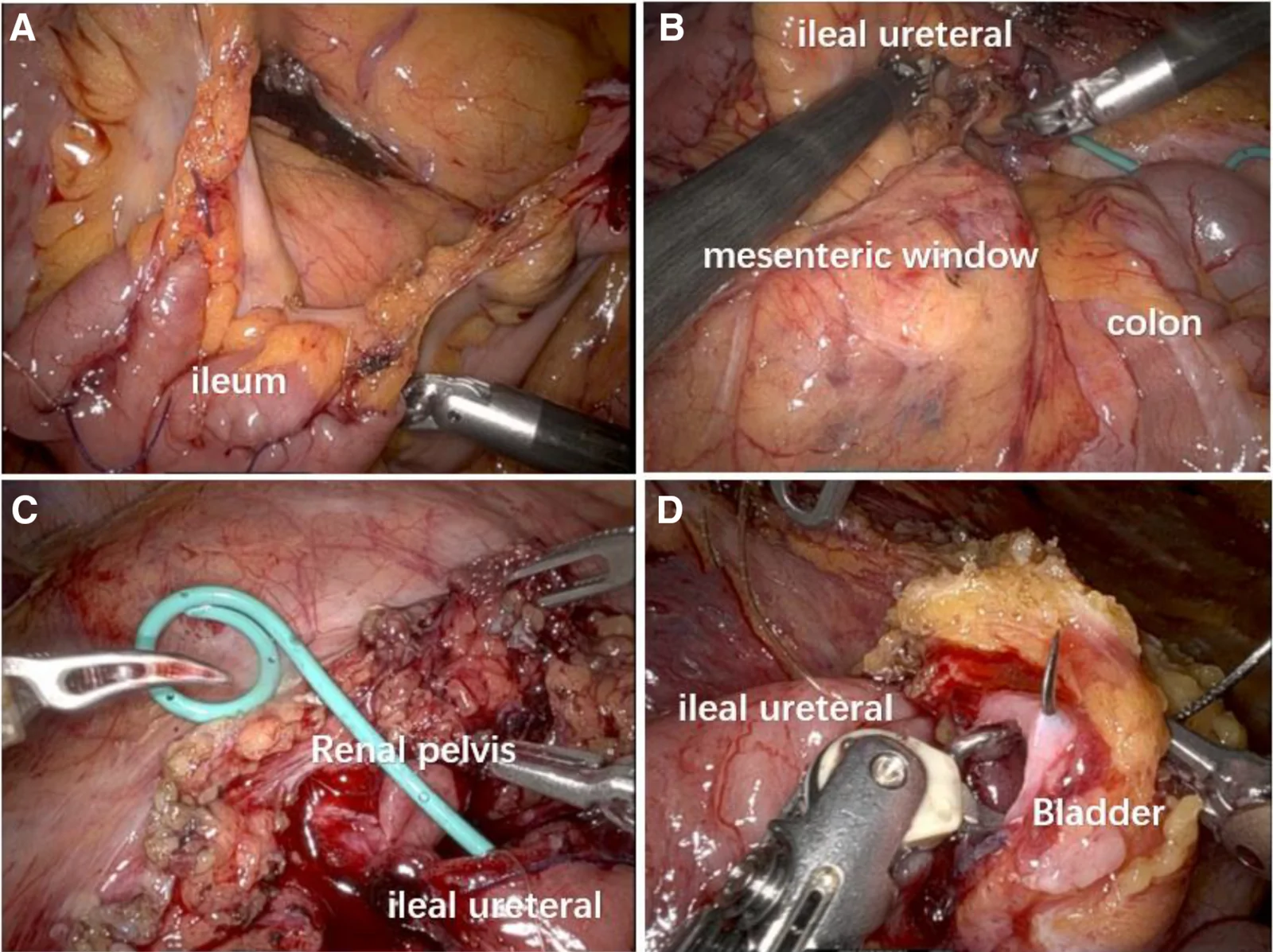
Completely intracorporeal robotic laparoscopic ileal ureter replacement. (A) The ileal segment was harvested for ureteral replacement, followed by reestablishment of intestinal continuity. (B) The ileal ureter was transposed retroperitoneally through a mesenteric window. (C) Ileal-renal pelvis anastomosis was performed. (D) Ileal-bladder anastomosis was performed.

The postoperative management protocol was as follows: The nephrostomy tube was clamped, and the urethral catheter was removed at 2 weeks postoperatively. The double-J stent was removed at 2 months postoperatively. The nephrostomy tube was ultimately withdrawn after confirming patency of the ileal ureter and absence of urinary extravasation on imaging.

Follow-up: Long-term postoperative follow-up revealed stable total renal function and satisfactory drainage of the reconstructed urinary tract in both patients (Fig. 4). Split renal function was not reassessed by dynamic renal scintigraphy; therefore, normal differential renal function cannot be claimed. To date, serum creatinine levels were 85.9 (case A) and 67.5 μmol/L (case B). No serious adverse events, including delayed anastomotic stricture, urinary reflux, recurrent infection, metabolic acidosis, or intestinal complications, were observed; only mild discomfort at the abdominal surgical site was noted. Subsequent surveillance comprised urinary tract imaging performed every 3 months during the 1st postoperative year, every 6 months during the 2nd year, and annually thereafter. Surveillance also included serum creatinine/GFR, urine culture, electrolytes, and blood gas analysis, with renal scintigraphy performed when clinically indicated.

**Figure 4.**
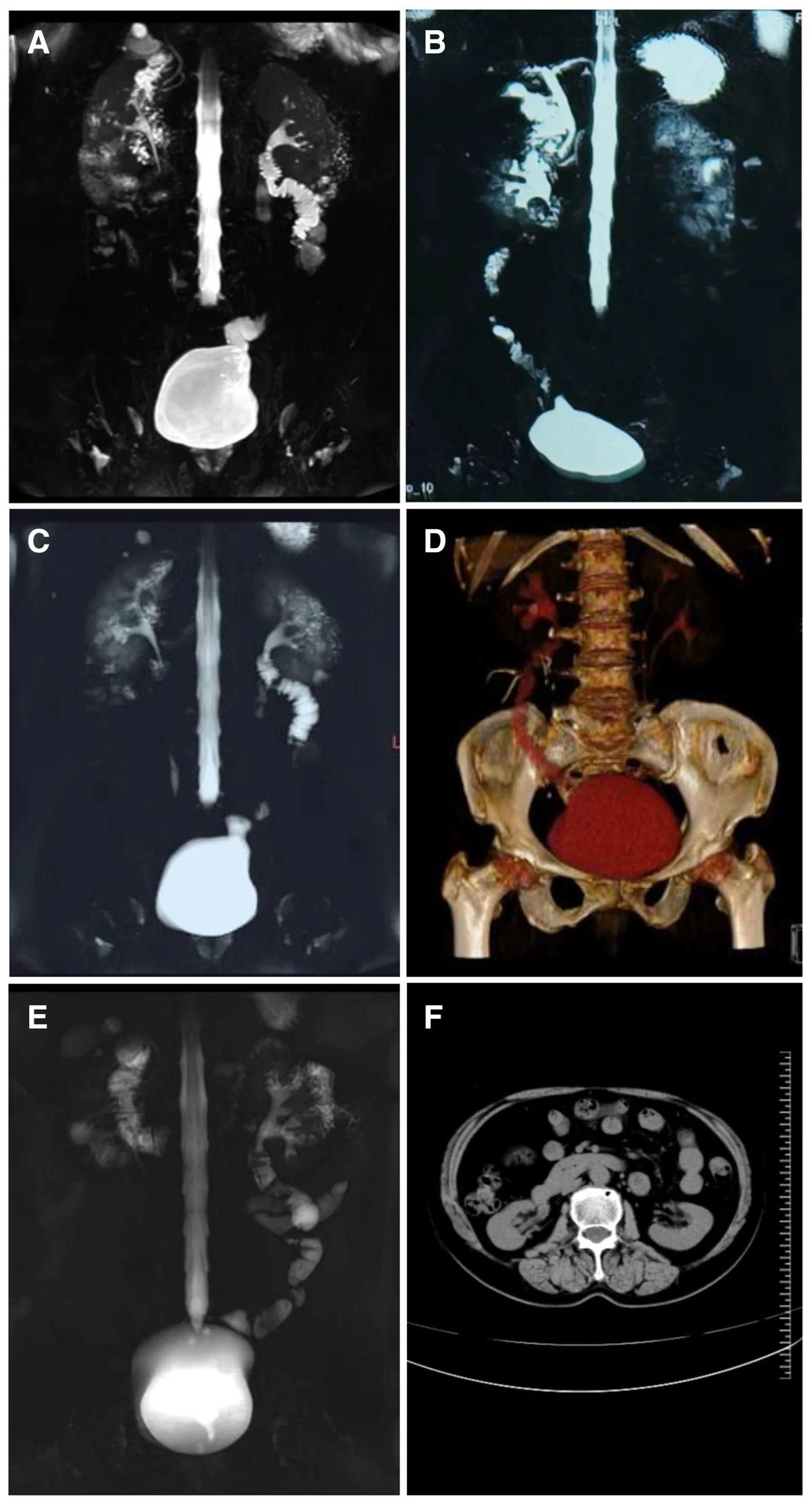
Postoperative follow-up imaging data were obtained at 3 months (A), 7 months (C), and 14 months (E) for case A, and at 3 months B), 7 months (D), and 16 months (F) for case B.

Due to the absence of formal medical records, the medical history was obtained solely from the patient’s verbal accounts. Consequently, potential limitations regarding the underlying mechanisms and detailed clinical information may exist in this report.

Written informed consent was obtained from both patients for the publication of their medical records, case details, and imaging data. The study was approved by the Ethics Committee of Gansu Provincial Hospital.

## 3. Discussion

Ureteroscopy is the most frequently employed minimally invasive technique for the management of upper urinary tract disorders. It is classified as a relatively simple and minimally invasive procedure because it does not require disruption of anatomical boundaries. However, it is important to recognize that minimally invasive procedures do not equate to low-risk procedures. In fact, the incidence of ureteroscopic intraoperative injury has been reported as high as 46.5%.^[1]^ Such injuries, including mucosal erosions, false passages, and perforations, may be overlooked due to their typically minor nature. Ureteral avulsion represents one of the most serious complications of ureteroscopy, with a prevalence ranging from 0.04% to 0.9%.^[2]^ Fortunately, the incidence of ureteral avulsion remains low despite a significant increase in the number of ureteroscopies.

The presumed mechanism leading to full-length ureteral avulsion is described below. Firstly, attributed to the tapered design of the ureteroscope, when the ureteroscope enters a ureter with a relatively small lumen, the application of excessive upward force will cause the proximal shaft of the ureteroscope to wedge into the ureter.^[3]^ This may result in the formation of a “scabbard” of ureter, as previously reported, and lead to the avulsion of the ureter from the bladder.^[4]^ Secondly, it is widely acknowledged that the UPJ is the most vulnerable to avulsion due to its comparatively thin wall structure.^[5]^ Inflammatory edema resulting from stone ingrowth, in conjunction with the damage to the ureteral mucosa caused by lithotripsy, serves to exacerbate the brittleness of the UPJ. Ultimately, operator inexperience and panic, compounded by suboptimal display of the field of view (poor return of the flushing fluid), precipitate a secondary avulsion of the UPJ upon the withdrawal of the ureteroscope.

More than a decade ago, the prevailing consensus was that nephrectomy of the affected side constituted the preferred response to full-length ureteral defects.^[4]^ This approach was favored due to its relative uncomplicatedness and safety, while ensuring a certain degree of life quality for patients. Nevertheless, many patients find this option unacceptable.

Renal autotransplantation was once regarded as the ideal solution for the treatment of full-length ureteral avulsion.^[6]^ However, the procedure requires an experienced kidney transplant team as a prerequisite. This may not be readily available in most hospitals, especially in emergency situations. Furthermore, the risk of graft loss has been documented to be as high as 17%, even in surgeries performed by a professional team.^[7,8]^

Pyeloureterostomy plus omentum investment outside the avulsed ureter and ureterovesical anastomosis can provide relatively satisfactory surgical outcomes.^[9]^ Nevertheless, the distal ureter is particularly susceptible to ischemic compromise and fibrosis, ultimately resulting in anastomotic stricture formation.^[10]^ In most emergencies, the reimplanted ureter may be performed as the initial intervention. Should renal deterioration be detected during postoperative surveillance, patients retain the option for secondary reconstructive procedures in an experienced center, such as ileal interposition, Boari flap, or autotransplantation,^[11]^ as demonstrated in case B.

Boari flap has been frequently reported in the literature as a surgical approach to long-segment ureteral avulsion. However, notable limitations include insufficient flap length, reduced postoperative bladder capacity, and anatomical constraints in right-sided applications.^[12]^

Reconstruction of the ureter using an intestinal segment was 1st reported by Schoemaker in 1911.^[13]^ Over the past decade, IUR has gained increasing recognition as a surgical intervention associated with a favorable prognosis and demonstrated absence of adverse effects on renal function. Although IUR may lead to complications such as intestinal obstruction, urinary stasis, hyperchloremic acidosis, and potential urointestinal malignancies, these risks can be effectively managed through standardized postoperative follow-up protocols.^[13]^ For totally intracorporeal laparoscopic ileal ureteral replacement, it was once considered a highly complex procedure, which led to its slow development. With the advancement of robotic surgery, a single-center study initiated by Yang et al^[14]^ demonstrated that totally intracorporeal RA-IUR exhibits favorable safety and efficacy in terms of operative duration, blood loss, recovery of intestinal function, and preservation of renal function.

RA-IUR in the 2 present cases was performed 5 months after the original injury. While early and delayed repair demonstrate comparable long-term outcomes,^[15,16]^ it is recommended that surgical intervention be performed at the earliest possible opportunity.^[2,17]^ This recommendation may be based on the premise of well-preserved vascularity at the site of injury and minimal retroperitoneal fibrosis, thereby reducing the complexity of the reconstructive procedure. However, it is probable that the operator’s inexperience results in a relatively small number of cases being repaired immediately. Based on our experience, in such cases, a nephrostomy should be performed as early as possible to reduce the risk of urinary extravasation. In addition, if there is local urinary extravasation or associated systemic infection, a nephrostomy should be performed on the affected side 1st, with surgical repair to be carried out after 3 to 6 months.

The ureter was completely lost in case A. In case B, since prior surgical repair had been undertaken, the local tissue was deemed nonviable, and ureteroneocystostomy was no longer considered feasible. As noted above, renal autotransplantation entails considerable risks of graft loss and bleeding, whereas the Boari flap offers only a limited length for ureteral substitution. However, lifelong and regular surveillance is required after RA-IUR to monitor for urinary tract obstruction, infection, metabolic complications, renal deterioration, and other relevant parameters.

In any event, the prevention of full-length ureteral avulsion merits the greatest attention. To mitigate the risk of full-length ureteral avulsion, a comprehensive preventive strategy should be implemented, encompassing preoperative, intraoperative, and technical considerations.^[18–21]^ The specific strategies are summarized in Table 1.

**Table 1:** Prevention strategies for full-length ureteral avulsion.

Preventive
1. Opting for small-caliber ureteroscopes
2. During the ureteroscopy procedure, operate gently and maintain a clear visual field
3. The ureteroscope surface should be adequately lubricated, especially when managing proximal stones, with lubricant applied along the entire shaft of the ureteroscope
4. If difficulty is encountered during ureteroscope advancement, initial placement of a double-J stent followed by a 2nd-stage procedure is generally safer^[9]^
5. If the ureteroscope becomes lodged at the ureteral orifice, a 2nd endoscope may be inserted into the bladder, and laser incision of the orifice can be performed^[4]^
6. Preoperative indwelling of a double-J stent for 1–2 weeks reduces the risk of ureteroscope incarceration^[11]^
7. Given the inherently delicate musculature of the proximal ureteral wall, stone retrieval basket deployment in the upper 3rd of ureter necessitates meticulous technique to avoid iatrogenic injury^[11]^
8. Ureteral inflammation (caused by etiological factors such as infection, tuberculosis, or neoplasms) constitutes a significant risk factor for ureteral avulsion^[18]^
9. Preoperative administration of ureteral smooth muscle relaxants^[19]^
10. Warm saline irrigation facilitates ureteral muscle relaxation and reduces the incidence of ureteral spasm^[20]^
11. Patients with prior SWL and/or corticosteroid therapy may have an elevated risk of ureteral avulsion^[21]^

SWL = shockwave lithotripsy.

## 4. Conclusion

Despite a substantial increase in ureteroscopy procedures, the incidence of complete ureteral avulsion remains remarkably low, underscoring the paramount importance of preventive strategies. This rare condition is currently treated on an individual basis, as there is no standardized approach. Irrespective of the therapeutic modality employed, continuous monitoring of upper urinary tract function (e.g., glomerular filtration rate) and anatomical architecture (e.g., pelvicalyceal system) is mandatory in lifelong management. For patients undergoing IUR, lifelong surveillance should specifically include monitoring for anastomotic or ureteral obstruction, recurrent urinary infection, metabolic acidosis and electrolyte abnormalities, urinary stasis or stones, and progressive renal deterioration.

## Author contributions

**Conceptualization:** Bin Feng.

**Data curation:** Xiaopeng Wu, Yilin Yuan, Yongtao He, Xuesong Li.

**Supervision:** Xuesong Li.

**Writing – original draft:** Kunlin Yang.**Writing – review & editing:** Bin Feng, Guanghui Yan.

## References

[1] Traxer O Thomas A. Prospective evaluation and classification of ureteral wall injuries resulting from insertion of a ureteral access sheath during retrograde intrarenal surgery. J Urol. 2013;189:580–4. 10.1016/j.juro.2012.08.197 22982421

[2] De Coninck V Keller EX Somani B. Complications of ureteroscopy: a complete overview. World J Urol. 2020;38:2147–66. 10.1007/s00345-019-03012-1 31748953

[3] Tanimoto R Cleary RC Bagley DH Hubosky SG. Ureteral avulsion associated with ureteroscopy: insights from the MAUDE database. J Endourol. 2016;30:257–61. 10.1089/end.2015.0242 26507706

[4] Ordon M Schuler TD Honey RJDA. Ureteral avulsion during contemporary ureteroscopic stone management: “the scabbard avulsion”. J Endourol. 2011;25:1259–62. 10.1089/end.2011.0008 21774664

[5] Perez Castro E Osther PJS Jinga V. Differences in ureteroscopic stone treatment and outcomes for distal, mid-, proximal, or multiple ureteral locations: the Clinical Research Office of the Endourological Society ureteroscopy global study. Eur Urol. 2014;66:102–9. 10.1016/j.eururo.2014.01.011 24507782

[6] Alizadeh M Valizadeh R Rahimi MM. Immediate successful renal autotransplantation after proximal ureteral avulsion fallowing ureteroscopy: a case report. J Surg Case Rep. 2017;2017:rjx028. 10.1093/jscr/rjx028 PMC5400459 28458835

[7] Vrakas G Sullivan M. Current review of renal autotransplantation in the UK. Curr Urol Rep. 2020;21:33. 10.1007/s11934-020-00986-z PMC7360532 32666391

[8] Yakupoglu YK Aki FT Kordan Y. Renal autotransplantation at three academic institutions in Turkey. Urol Int. 2016;97:466–72. 10.1159/000448482 27505010

[9] Zhong M-Z Huang W-N Huang G-X Zhang E-P Gan L. Long-term results of extended Boari flap technique for management of complete ureteral avulsion: a case report. World J Clin Cases. 2022;10:12610–6. 10.12998/wjcc.v10.i34.12610 PMC9791534 36579100

[10] Gao P Zhu J Zhou Y Shan Y. Full-length ureteral avulsion caused by ureteroscopy: report of one case cured by pyeloureterostomy, greater omentum investment, and ureterovesical anastomosis. Urolithiasis. 2013;41:183–6. 10.1007/s00240-012-0541-8 23503882

[11] Unsal A Oguz U Tuncel A. How to manage total avulsion of the ureter from both ends: our experience and literature review. Int Urol Nephrol. 2013;45:1553–60. 10.1007/s11255-013-0505-0 23884730

[12] Mauck RJ Hudak SJ Terlecki RP Morey AF. Central role of Boari bladder flap and downward nephropexy in upper ureteral reconstruction. J Urol. 2011;186:1345–9. 10.1016/j.juro.2011.05.086 21855904

[13] Kocot A Kalogirou C Vergho D Riedmiller H. Long-term results of ileal ureteric replacement: a 25-year single-centre experience. BJU Int. 2017;120:273–9. 10.1111/bju.13825 28220579

[14] Yang K Wang X Xu C. Totally intracorporeal robot-assisted unilateral or bilateral ileal ureter replacement for the treatment of ureteral strictures: technique and outcomes from a single center. Eur Urol. 2023;84:561–70. 10.1016/j.eururo.2023.04.022 37225525

[15] Al-Awadi K Kehinde EO Al-Hunayan A Al-Khayat A. Iatrogenic ureteric injuries: incidence, aetiological factors and the effect of early management on subsequent outcome. Int Urol Nephrol. 2005;37:235–41. 10.1007/s11255-004-7970-4 16142549

[16] Ding G Li X Fang D Hao H Li X Zhou L. Etiology and ureteral reconstruction strategy for iatrogenic ureteral injuries: a retrospective single-center experience. Urol Int. 2021;105:470–6. 10.1159/000511141 33744882

[17] Chalya PL Massinde AN Kihunrwa A Simbila S. Iatrogenic ureteric injuries following abdomino-pelvic operations: a 10-year tertiary care hospital experience in Tanzania. World J Emerg Surg. 2015;10:17. 10.1186/s13017-015-0011-z PMC4359460 25774212

[18] Hosseini SR Gorji A Fakhr Yasseri AM. Endourological management of mucosal ureteral avulsion: case report. Urol Case Rep. 2021;36:101595. 10.1016/j.eucr.2021.101595 PMC7878988 33614411

[19] Adhoni MZU Haider M Somani B. Does silodosin improve primary ureteroscopy access and outcomes: meta-analysis of randomized controlled trials. J Endourol. 2024;38:1148–55. 10.1089/end.2024.0413 39180290

[20] Mohammadzadeh Rezaei MA Akhavan Rezayat A Tavakoli M Jarahi L. Evaluation the result of warm normal saline irrigation in ureteral endoscopic surgeries. Urol J. 2018;15:83–6. 10.22037/uj.v0i0.3872 29250762

[21] Sevinc C Balaban M Ozkaptan O Yucetas U Karadeniz T. The management of total avulsion of the ureter from both ends: our experience and literature review. Arch Ital Urol Androl. 2016;88:97. 10.4081/aiua.2016.2.97 27377083

